# Molecular Epidemiology and Clinical Characteristics of an Outbreak on Respiratory Virus Coinfection in Gansu, China

**DOI:** 10.3390/v16040540

**Published:** 2024-03-30

**Authors:** Wu Liu, Hui Zhang, Tianshuo Zhao, Xianming Cai, Liguo Yang, Genxia Gao, Xiaoyan Che, Zhenhong Zhu, Tongxia Zeng, Fuqiang Cui

**Affiliations:** 1 Jingyuan County Center for Disease Control and Prevention, Jingyuan 730699, China; 13893030697@163.com (W.L.); 15109432095@163.com (L.Y.); 13884267412@163.com (G.G.); 18394130803@163.com (X.C.); 19809431872@163.com (Z.Z.); 2 Gansu Provincial Center for Disease Control and Prevention, Key Laboratory of Pathogenic Microbiology and Immunology (Gansu Province), Lanzhou 730050, China; xxwandzh@163.com; 3 Department of Health Inspection, Vaccine Research and Evaluation Center, School of Public Health, Peking University, Beijing 101100, China; zts2018@pku.edu.cn (T.Z.); 1710306124@pku.edu.cn (X.C.); 4 Scientific Research Training Department, Baiyin City Center for Disease Control and Prevention, Baiyin 730900, China

**Keywords:** coinfection, human metapneumovirus, human rhinovirus, outbreak, respiratory syncytial virus

## Abstract

This study aims to analyze the epidemiological and pathogenic characteristics of an outbreak primarily caused by respiratory syncytial virus (RSV), human rhinovirus (HRV), and human metapneumovirus (HMPV) in a kindergarten and primary school. The outbreak was investigated by field epidemiological investigation, and the common respiratory pathogens were screened by RT-PCR detection technology. The attack rate of this outbreak was 63.95% (110/172). Main symptoms included cough (85.45%), sore throat (60.91%), and sneezing (60.00%). Multifactorial logistic regression analysis revealed that continuous handwashing and mouth and nose covering when sneezing were protective factors. All 15 collected throat swab specimens tested positive for viruses, with HMPV as the predominant pathogen (80.00%), followed by HRV (53.33%), and two cases of positive respiratory syncytial virus (13.33%). Among them, six samples showed coinfections of HMPV and HRV, and one had coinfections of HMPV and RSV, resulting in a coinfection rate of 46.67%. Genetic sequencing indicated that the HMPV genotype in this outbreak was A2c, and the HRV genotype was type A, resulting in a coinfection outbreak of HMPV, HRV, and RSV in schools and kindergartens, suggesting that multi-pathogen surveillance of respiratory tract infections should be strengthened.

## 1. Introduction

Respiratory tract infections are prevalent infectious diseases, with a significant impact on infants, the elderly, individuals with chronic illnesses, hematopoietic stem cell transplant recipients, and immunocompromised individuals, as evidenced by their ranking among the top five causes of global mortality in 2015 [1,2]. Viruses are the predominant pathogens responsible for acute respiratory infections in children, primarily transmitted through air, droplets, and close contact [3]. Given that schools serve as significant gathering places for children, they are susceptible to becoming hotspots for the transmission of respiratory infections.

Severe outcomes such as hospitalization and mortality in cases of infection are predominantly associated with viral respiratory infections. Notably, respiratory syncytial virus (RSV), human rhinovirus (HRV), and human metapneumovirus (HMPV) are significant pathogens responsible for acute respiratory infections across all age groups globally [4,5]. RSV, first isolated from children in 1957, is considered the most critical virus causing acute respiratory infections in children [6]. HRV, discovered in the 1950s, is a causative agent for over half of common cold-like illnesses [7,8]. HRV is commonly associated with the common cold and mild upper respiratory tract infections, but it can also cause severe respiratory infections in the immunocompromised or those with chronic lung diseases [9,10]. These three viruses share high similarities in transmission characteristics and clinical symptoms, leading to a spectrum of diseases ranging from mild upper respiratory infections to severe lower respiratory infections in children and adults alike [4]. The majority of infants and children have been infected with RSV, HMPV, and HRV, leading to prevalent diseases such as bronchiolitis and pneumonia. This trend persists in adults, where almost all individuals show serological evidence of prior infections with RSV, HMPV, and HRV [11,12,13]. While healthy adults typically experience self-limiting symptoms following infection, individuals with compromised immune systems are at an elevated risk of developing severe illnesses after being infected with RSV, HMPV, and HRV [9,14,15]. Due to insufficient attention given to this population, they contribute significantly to the global burden of hidden diseases each year. 

Gansu Province in the northwest of China displays a wide range of climatic conditions, encompassing high-altitude mountainous areas, desert grasslands, and temperate semi-humid regions. Its geographical location, bordering multiple provinces, may impact population movements from other areas and consequently influence the speed and extent of epidemic spread. In contrast to the eastern coastal provinces of China, Gansu Province exhibits a relatively lower population density. Lower population density implies reduced inter-community contact, but it may also lead to the dispersed distribution of medical resources, presenting challenges for epidemic control. The respiratory infection epidemic simultaneously occurred in adjacent schools and kindergartens. Long-term monitoring data from the Gansu Provincial Center for Disease Control and Prevention indicate a different incidence pattern compared to previous reports, prompting special investigations. This research aims to comprehensively analyze the epidemiological features and pathogen characteristics of the epidemic.

Through an in-depth examination of this epidemic, we aim to better understand the transmission mechanisms of respiratory infections in closed environments, providing scientific evidence for developing more effective prevention and control strategies in the future. The outcomes of this study will contribute to a deeper understanding of the transmission mechanisms of respiratory infections, offer robust support for responding to similar events in the future, and serve as a reference for the prevention and control of respiratory outbreaks in collective settings.

## 2. Materials and Methods

### 2.1. Overview of the Epidemic

This epidemic occurred in a local kindergarten and primary school, consisting of 9 grades and nine classes. The educational institutions had a combined population of 140 students and children, and 32 staff members. Among these were 84 primary school students, 23 primary school staff, 56 kindergarten children, and 9 kindergarten staff. Both schools operated as day schools, occupying spacious buildings with classrooms. During school hours, doors and windows could be opened for ventilation, and no healthcare physicians were on-site. The schools did not provide meals or accommodation, and they were approximately 400 m from residential buildings. Students commuted daily between school and home on foot, escorted by parents. On 25 May 2023, the kindergarten reported that more than 20 students were absent due to illness, and the primary school reported that more than 10 students in a class were absent due to illness, with the main symptoms being cough, fever, sore throat, etc. The county CDC immediately entered the scene to investigate, including the basic information about the patients, the incidence, epidemiological characteristics, respiratory symptoms, signs, and so on. In running a case search on suspicious patients between 1 May and 15 June, 101 cases meeting the case definition were identified in the two institutions, affecting children, students, and staff, resulting in an overall attack rate of 72.14% (101/140). The highest attack rate was observed in the kindergarten’s small class, reaching 92.86% (13/14), while the staff’s attack rate was 28.13% (9/32).

### 2.2. Study Subjects

All kindergarten children, primary school students, and staff with or without respiratory symptoms between 1 May and 15 June 2023, in kindergartens and primary schools within a township of Gansu Province were included as subjects. The case definitions were as follows: (a) Suspected cases: Individuals who exhibited coughing, phlegm production, sore throat, runny nose, or other respiratory clinical symptoms lasting more than one day. They may also experience fatigue, fever (axillary temperature ≥ 37.3 °C), headache, limb pain, or other systemic symptoms lasting more than one day. (b) Confirmed cases: Suspected cases whose throat swabs tested positive for respiratory virus nucleic acid through Reverse Transcription Polymerase Chain Reaction (RT-PCR). (c) Control group: Kindergarten children, primary school students, and staff who had not shown any signs of respiratory tract infection in the past 45 days within kindergartens and primary schools in a township of Gansu Province were selected as the healthy control group through investigation.

### 2.3. Outbreak Investigation

The investigation involved searching for cases through local medical records, interviews with kindergarten caregivers and teachers, and on-site and telephone follow-ups with parents. A standardized questionnaire covering basic information, illness details, clinical data, influenza vaccination status, contact history, and risk factors was designed and administered to each class. Both the kindergartens and primary schools implemented a daily epidemic reporting system.

A comprehensive survey was conducted on the overall layout of kindergartens and primary schools, children’s home living conditions, environmental hygiene, hygiene facilities in kindergarten bathrooms, handwashing facilities, and disinfection status of two locations. Parents’ and children’s travel history and participation in extracurricular activities in the last seven days were also investigated.

On-site questionnaires were conducted for parents of affected kindergarten children, caregivers, and class teachers. Controls were randomly selected within the class, meeting the criteria of no corresponding respiratory clinical symptoms from 1 May to 15 June 2023. The investigation of case and control groups in kindergartens and primary schools was conducted based on the questionnaire content.

Local Centers for Disease Control (CDC) epidemiological investigators underwent unified training, adhering to standardized questionnaire understanding. The questionnaires were cross-checked by designated personnel, and any missing or incomplete items were supplemented through telephone follow-ups. Double-entry of questionnaire data was performed, and consistency checks were conducted on the entered results.

### 2.4. Sample Collection and Pathogen Detection

Throat swab samples of suspected cases were collected by qualified medical personnel, and case data were collected and stored in a non-inactivated sampling tube containing 3–4 mL sterile sampling solution (Yocon Biotechnology (Beijing) Co., Ltd., Beijing, China, Batch number: 01230319). Nasal swab samples were collected, and the novel coronavirus (2019-nCoV) antigen detection was performed using the colloidal gold method on site (Shanghai Biogerm Medical Technology Co., Ltd., Shanghai, China, batch number 20221109A). The sampling and detection procedures were conducted per the kit instructions. Throat swab samples were collected and stored at 4 °C within 24 h and transported to the Virus Laboratory of Gansu Provincial Center for Disease Control and Prevention. Nucleic acid was extracted from pharyngeal swab samples of 15 cases by the magnetic bead method (Guangzhou Daan Gene Co., Ltd., Guangzhou, China, article No: DA1003), and the extraction process was carried out strictly following the kit instructions. RT-PCR method was used to detect six respiratory viruses (Shanghai Berger Medical Technology Co., Ltd., Shanghai, China, article No.: SJ-HX-807-2): respiratory syncytial virus (RSV), parainfluenza virus I (PIV-I), parainfluenza virus III (PIV-III), human rhinovirus (HRV), adenovirus (ADV), and human metapneumovirus (HMPV). The experimental methods and results were interpreted per the kit instructions.

### 2.5. Amplification and Genotype Identification of Target Genes

The pathogen detection results showed mainly HMPV and HRV infection, so the HMPV- and HRV-positive samples were further identified. RT-PCR was used to amplify the G gene in HMPV-positive samples, and the VP1 region was identified in HRV-positive samples. The Measles Room provided the identification primer, the National Institute of Viral Diseases, Chinese Center for Disease Control and Prevention, and the amplification reagent was used with One Step RT-PCR kit (TAKARA, Tokyo, Japan, article number: DRR057A). Sanger sequencing was used on PCR amplification products. BLAST online through https://blast.ncbi.nlm.nih.gov/Blast.cgi (accessed on 25 May 2023). was used to obtain the nucleotide sequence alignment sequence and determine the most similar sequences. Regarding the literature from Genbank (https://www.ncbi.nlm.nih.gov/) (accessed on 25 May 2023), we downloaded RSV for each genotype reference sequence through MEGA7.0 software, together with the above sequence-building related evolutionary tree.

### 2.6. Statistical Analysis

EpiDate3.1 established the database, and SPSS 19.0 was used for statistical analysis. Descriptive epidemiological analysis methods were employed to illustrate the temporal, spatial and population distribution of the outbreak, and logistic regression models were applied to analyze factors influencing the outbreak for the case–control studies (comparison between 110 cases and 64 controls). A significance level of α = 0.05 (two-tailed) was used, with *p* < 0.05 indicating statistical significance.

## 3. Results

The affected kindergarten and primary school relocated to the current residential building in 2018. The kindergarten is adjacent to the primary school, with classrooms covering an area of 90 m^2^. The average space per person is 2 m^2^, including coatrooms and bathrooms. The bathroom facilities are complete, and no windows are directly connected to the outdoors, ensuring a well-maintained indoor environment. The primary school classrooms have an area of 72 m^2^, offering good ventilation. Each floor is equipped with two separate bathrooms for males and females. Although the bathrooms have windows, they are kept closed. The handwashing facilities are complete, but hand soap is not provided. Most faucets are not functioning correctly. The primary school offers teachers bottled, purified water while students bring their own water. The kindergarten, on the other hand, centrally supplies purified water for drinking.

In the outbreak, 110 cases of acute upper respiratory infections were reported, resulting in a morbidity rate of 63.95% (110/172). All cases were mild, with no reports of severe or fatal cases. The main symptoms included coughing (85.45%), sore throat (60.91%), sneezing (60.00%), nasal congestion (54.55%), runny nose (54.55%), fever (53.64%), and fatigue (53.64%), among others, as detailed in Table 1.

### 3.1. Epidemiological Characteristics

#### 3.1.1. Temporal Distribution

**Figure 1:**
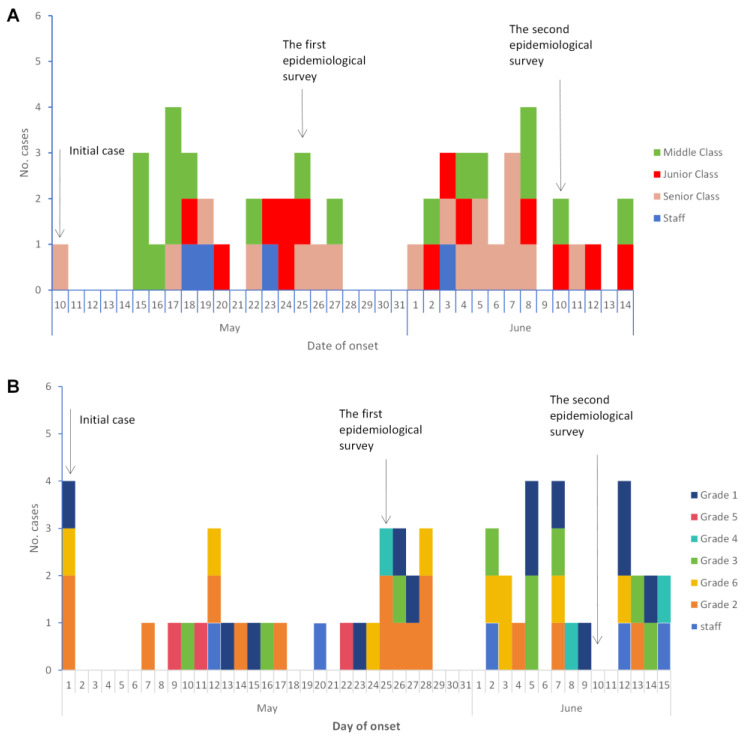
Time distribution of febrile respiratory syndrome cases in kindergartens/primary schools in Gansu Province in 2023. (**A**) The cases in kindergartens; (**B**) the cases in primary schools.

The first case in the kindergarten occurred on 10 May, affecting a preschooler in the senior class, and the last case appeared on 14 June, spanning 36 days. The epidemic exhibited a bimodal distribution, with peaks on 17 May and 8 June, consistent with the characteristics of intergenerational transmission in respiratory infectious diseases. The value-added pattern revealed five generations of cases (Figure 1A).

The first case emerged on 1 May; subsequent generations occurred every 7 to 8 days. A similar pattern was observed in the primary school, with the first case on 1 May and a 46-day duration. The epidemic showed a sawtooth distribution, and five generations appeared, following a similar pattern of intervals (Figure 1B).

#### 3.1.2. Spatial Distribution

Cases were distributed across various classes in both the primary school and kindergarten. The primary school had 57 cases with a morbidity rate of 53.27% (57/107), while the kindergarten had 53 cases with a morbidity rate of 81.54% (53/65). The difference in morbidity rates between the kindergarten and primary school was statistically significant (χ^2^ = 14.016, *p* < 0.05). Morbidity rates varied significantly among different grades in the primary school, ranging from 18.75% (3/16) in the fifth grade to 86.67% (13/15) in the first grade (χ^2^ = 19.095, *p* < 0.05). The kindergarten showed no significant difference in morbidity rates between classes (χ^2^ = 1.131, *p* > 0.05). Table 2 provides further details. The analysis indicated clustering in the distribution of cases in both kindergarten and primary school classes (χ^2^ = 13.83, *p* < 0.01; χ^2^ = 268.38, *p* < 0.01). Intra-family relationships, especially among siblings, contributed to a significant contact history between different classes within the same school. Out of 44 cases from 20 families, family clustering constituted 40% (44/110) of the total cases.

#### 3.1.3. Population Distribution

Out of the 110 cases, students accounted for 101, resulting in a morbidity rate of 72.14% (101/140). Faculty members experienced nine cases, with a morbidity rate of 28.13% (9/32). The difference in morbidity rates between students and faculty was statistically significant (χ^2^ = 57.607, *p* = 0.000). Kindergarten children had a morbidity rate of 87.50% (49/56), while faculty had a rate of 44.44% (4/9), with a significant difference (adjusted χ^2^ = 6.903, *p* = 0.009). In the primary school, student morbidity was 61.90% (52/84), and faculty morbidity was 21.74% (5/23), also showing a significant difference (χ^2^ = 11.702, *p* = 0.001). Gender-wise analysis revealed no significant difference in morbidity rates between males and females in both the kindergarten and primary school (Table 2).

### 3.2. Case–Control Study

A total of 174 questionnaires were collected, with a response rate of 100.00% (110 cases, 64 controls) after excluding two duplicate questionnaires. Variables such as infection status, population category, history of COVID-19 infection, contact with flu-like cases in the last seven days, location of contact, occupation, age, handwashing duration, covering mouth and nose when sneezing, immediate handwashing after sneezing, immediate handwashing after class, touching lips/fingers, eye-rubbing habits, cleanliness of hands and nails, use of transportation to school, time spent in public places (excluding school), family members covering mouth and nose when sneezing, home ventilation status, preventive medication, sleep time, frequency of colds in the past six months, and participation in extracurricular training were considered as independent variables. After adjusting for confounding factors such as gender, multiple logistic regression analysis showed that handwashing for 10–30 s or >30 s and family members covering mouth and nose when sneezing were protective factors (OR = 0.06, 95.0% CI = 0.01–0.51, *p* = 0.01; OR = 0.06, 95.0% CI = 0.01–0.34, *p* = 0.00; OR = 0.23, 95.0% CI = 0.06–0.95, *p* = 0.04). Factors such as contact with flu-like cases in the last seven days, walking to and from school, window ventilation (<2 h), frequency of colds (≥3 times or = 2 times), and participation in extracurricular training were identified as risk factors for the outbreak, as shown in Table 3 (OR = 1.18, 95% CI = 1.05–1.60, *p* = 0.01; OR = 13.47, 95.0% CI = 1.92–94.75, *p* = 0.01; OR = 27.99, 95.0% CI = 1.33–589.54, *p* = 0.03; OR = 424.73, 95.0% CI = 22.36–8066.18, *p* < 0.01; OR = 18.04, 95.0% CI = 4.80–67.74, *p* < 0.01; OR = 32.55, 95.0% CI = 3.16–335.29, *p* < 0.01). 

### 3.3. Laboratory Examination

**Figure 2:**
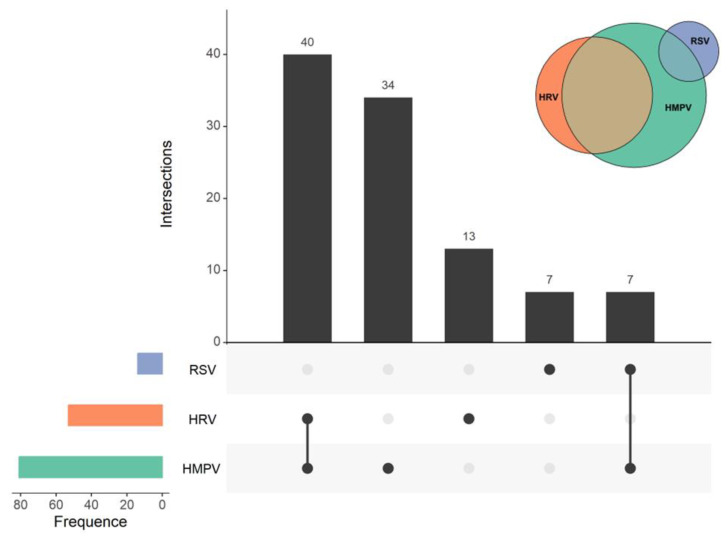
Coinfection and detection of respiratory viruses. RSV infection is indicated by blue, HRV by orange, and HMPV by green. The overall detection of each pathogen is displayed in the lower left corner, while the combined and single pathogen detections are shown in the upper right corner.

The results of 15 nasal swab samples tested in the field were negative for 2019-nCoV antigen. The results of 15 nasopharyngeal swab samples sent to the laboratory of the Gansu Provincial Center for Disease Control and Prevention were positive for HMPV (80.00%, 12/15), followed by HRV (53.33%, 8/15) and RSV (13.33%, 2/15) in 2 cases. Among these, six samples exhibited coinfections of HMPV and HRV, and one sample showed a combination of HMPV and RSV, resulting in a coinfection rate of 46.67% (Figure 2).

**Figure 3:**
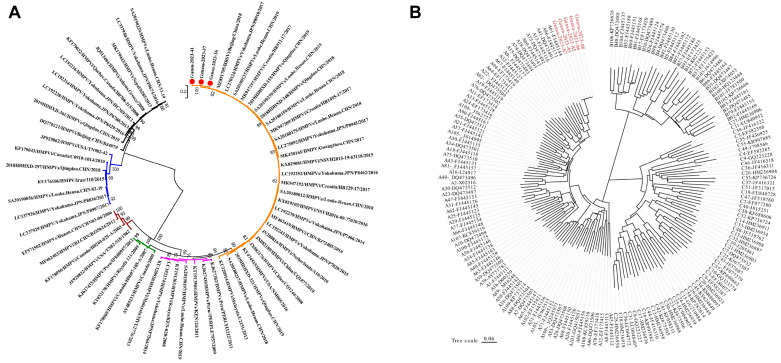
The phylogenetic tree of human metapneumovirus (HMPV) strains and human rhinovirus (HRV). (**A**) The phylogenetic tree of HMPV strains is based on G genes (**B**). The phylogenetic tree of HRV strains is based on VP1 genes.

Sanger sequencing revealed three complete HMPV G gene sequences, designated as Gasu-2023-36, Gasu-2023-37, and Gasu-2023-41. Blast comparisons on GenBank classified them within the A2c sub-lineage. The three HMPV sequences were used to construct a phylogenetic tree with 59 HMPV reference strains from around the globe, as shown in Figure 3A, indicating the A2c genotype for HMPV in this outbreak. Additionally, five complete HRV VP1 gene sequences were obtained, named Gasu-2023-A3, Gasu-2023-A5, Gasu-2023-A6, Gasu-2023-A7, and Gasu-2023-A8. Blast comparisons on GenBank confirmed these as HRV-A type. A phylogenetic tree in Figure 3B illustrates the HRV infection in this outbreak as type A. Due to low viral load, gene typecasting could not be performed because an RSV sequence was not obtained in this study.

## 4. Discussion

The common viruses that can cause human respiratory tract infection are influenza virus (flu), respiratory syncytial virus (RSV), parainfluenza virus (PIV), rhinovirus (HRV), human metapneumovirus (HMPV), adenovirus (ADV), etc., among which there are also mixed infections [16,17]. Due to the challenge of establishing lasting immunity post-respiratory virus infection, populations are generally susceptible, with transmission intensifying in densely populated areas, resulting in outbreaks. Numerous studies report school outbreaks caused by HMPV, HRV, or RSV [18,19,20,21,22,23]. In this paper, the epidemiological investigation and etiology results of an upper respiratory tract infection outbreak in a kindergarten and primary school in Jingyuan County, Baiyin City, Gansu Province, were summarized and analyzed. We used the RT-PCT technique to type HRV genotypes, which has been proven effective when differentiating HRV from enteroviruses and human parechoviruses with close genetic relatedness [24,25,26]. The outbreak was a mixed infection of HMPV + HRV, HMPV + RSV, and an outbreak caused by RSV infection. In Gansu Province and Yinchuan City of Ningxia Hui Autonomous Region, cases of HMPV + HRV coinfection were found in the detection of viruses in hospitalized children. Still, cases of HMPV and RSV coinfection were rarely reported [27,28]. Studies have shown that HMPV and RSV are the primary pathogens of lower respiratory tract infections in children, often affecting infants under two years of age; in particular, children under six months of age frequently present with bronchopneumonia, the main clinical manifestations of which are fever, cough, wheezing, shortness of breath, vomiting, runny nose, sore throat, headache, etc. [18,19,20,21,22,23]. Conversely, HMPV infections in adults are often asymptomatic [19]. Monitoring data from Gansu suggests that HRV infections primarily affect young children [27], resulting in symptoms of lower respiratory tract infections, with HRV-positive children exhibiting more pronounced fever and vomiting symptoms [28]. HRV infections are associated with a higher risk of other diseases, often leading to respiratory symptoms such as fever and cough [28]. This study reveals that coinfections of HMPV, HRV, and RSV not only cause illnesses in children but also pose a risk to adult staff. Clinical presentations of cases in this outbreak primarily featured fever accompanied by respiratory symptoms, similar to cases caused by single HMPV, RSV, or HRV infections [29,30,31,32,33,34]. Additionally, the outbreak exhibited flu-like symptoms. The duration of the outbreak in the two schools was 36 and 46 days, longer than acute respiratory infection outbreaks caused by single infections of HMPV, RSV, or HRV [29,30,31,32,33,34]. HMPV and RSV infections typically show seasonal patterns, with higher prevalence in winter and spring [20,32]. In contrast, HRV infections exhibit a more noticeable seasonal peak in autumn [29], being the most common virus causing common colds in the summer and autumn. This outbreak, involving a mixture of HMPV, RSV, and HRV, occurred in May, with a deviation in the seasonal prevalence of HMPV and RSV and an early trend for HRV infections. The prolonged and unclear seasonal patterns suggest a multi-source exposure for this outbreak. Due to sibling relationships, the outbreak occurred in a primary school with significant contact history among different classes and schools. The cases demonstrated apparent class clustering, with higher attack rates in younger classes, particularly in the first grade and kindergarten, where students and preschoolers had higher attack rates than teaching staff.

Multiple logistic regression analysis revealed that, when compared to the control group and after adjusting for factors that may affect the continued spread of the epidemic, contact with flu-like cases within seven days, walking to and from school, window ventilation (<2 h), frequency of colds (≥3 times or =2 times), and participation in extracurricular training were identified as significant risk factors for the continued spread of the outbreak. These risk factors played an essential role in the sustained transmission of the epidemic. Contact with flu-like cases within seven days may lead to inhaling virus aerosols from infected individuals. Walking to and from school on a single road increased contact opportunity, and participation in extracurricular training may contribute to the spread between schools and grades. A higher frequency of colds in the past six months (≥3 times or =2 times) may indicate a weakened immune system due to repeated infections with COVID-19 or HRV, making individuals more susceptible to viral infections. Conversely, handwashing for 10–30 s or >30 s and family members covering their mouth and nose when sneezing were identified as protective factors. Handwashing and covering the mouth and nose when sneezing are effective measures to prevent respiratory infections and reduce the spread of viruses. Therefore, kindergartens, primary schools, and other crowded places should establish a working mechanism for the prevention and control of infectious diseases, the implementation of morning and afternoon inspection systems, and the follow-up of children absent due to illness through the morning and afternoon inspection so that children with abnormal symptoms are found in time and isolated for observation and treatment as soon as possible, or taken to the hospital for treatment. At the same time, it is essential to publicize the health knowledge of respiratory infectious diseases in schools and kindergartens, improve the personal protection awareness of students and staff, improve behavioral habits, and often ventilate classrooms and families, which can effectively reduce the occurrence of clustered epidemics. Timely reporting of outbreaks and continuous monitoring and research by disease control and medical institutions on the variation and evolution of infectious disease pathogens provide crucial data support for developing preventive and control strategies for respiratory pathogens such as HMPV, HRV, and RSV in the local area. 

This outbreak identified 12 HMPV-positive samples, obtaining four complete sequences. Evolutionary tree analysis indicated that the HMPV in this outbreak belonged to the A2c genotype; it was most similar to the circulating strains in Beijing, Qingdao, and Henan (95.75–96.64%), among which the full gene sequence homology with the sequencing strains in Beijing 2018 and Qingdao 2019 reached 100%. The first strain of the HMPV A2c genotype was discovered in Okinawa, Japan in 2011 [35], with similar strains reported in China [36]. This outbreak identified eight HRV-positive samples, obtaining five complete VP1 region sequences. VP1 region sequencing revealed that the HRV in this outbreak belonged to species A, and evolutionary tree analysis suggested that the strains were most similar to those prevalent in Fujian (97.03–97.15%). Evolutionary tree analysis closely resembles prevalent strains in Fujian and other regions. This outbreak occurred in schools and kindergartens in a newly established immigrant community, with residents engaged in work from other areas. Based on epidemiological investigations and laboratory genetic sequencing results, the outbreak was caused by mixed infection of HMPV, HRV, and RSV viruses in local schools and kindergartens. The high genetic homology of the HMPV A2c strain suggests possible importation from Beijing, Qingdao, Henan, and other areas. Similarly, the high genetic homology of the HRV A strain suggests possible importation from Fujian and other areas. Both HMPV A2c and HRV A strains are currently dominant circulating strains. Further research is needed to investigate whether the combined infection with RSV increases pathogenicity and invasiveness compared to single infections, as well as to elucidate the mechanisms of triple infection. However, there are still some limitations to this study. Firstly, the outbreak occurred in northwest China and therefore the results may not be generalizable to other regions due to variations in climate, temperature, humidity, and human immune response following COVID-19 infection. Secondly, samples were not collected and tested for each case, which hindered accurate determination of pathogen type and corresponding clinical manifestations. Additionally, the long time interval between infection and the survey as well as inaccurate recall by some participants may have introduced bias into the analysis results. Finally, while RT-PCR was employed for virus identification in this study, critical values for experimental quantification were not utilized to confirm active infections. In conclusion, this paper presents preliminary evidence of mixed HMPV, HRV, and RSV infections in schools and kindergartens, but further research is warranted.

## Figures and Tables

**Table 1:** Main clinical manifestations of febrile respiratory syndrome in a township of Gansu Province in 2023.

Symptoms	No. Cases = 110	Proportion (%)
Cough	94	85.45
Sore throat	67	60.91
Sneezing	66	60
Nasal congestion	60	54.55
Runny nose	60	54.55
Fever (°C)	59	53.64
Low-grade fever (37.5–38.0)	8	7.27
Moderate fever (38.1–39.0)	44	40
High fever (≥39.1)	7	6.37
Fatigue	59	53.64
Loss of appetite	57	51.82
Wheezing	46	41.82
Enlarged tonsils	35	31.82
Abdominal pain	34	30.91
Headache	30	27.27
Nausea	30	27.27
Chills	27	24.55
Phlegm production	22	20
Vomiting	20	18.18
Muscle aches	13	11.82
Diarrhea	9	8.18

**Table 2:** Grade distribution of febrile respiratory syndrome cases in a township of Gansu Province in 2023.

School	Class	Class Size		No. Cases		Incidence (%)
		Male	Female	Male	Female	
Primary School	Grade 1	8	7	8	5	86.67
	Grade 2	8	12	4	11	75.00
	Grade 3	8	5	3	5	61.54
	Grade 4	3	4	2	1	42.86
	Grade 5	7	9	1	2	18.75
	Grade 6	6	7	4	5	69.23
	Teaching Staff	16	7	5	1	26.09
Kindergarten	Junior Class	9	5	9	4	92.86
	Middle Class	11	9	9	9	90.00
	Senior Class	7	15	7	11	81.82
	Teaching Staff	4	5	1	3	44.44

**Table 3:** Multivariate logistic regression analysis of respiratory virus coinfection.

Variable	β	SE	Wald	*p*	OR	95% CI	
						Inferior	Superior
COVID-19 infection history (2)	0.67	0.68	0.97	0.32	1.95	0.52	7.33
Contact with flu-like cases in the past seven days (2)	1.71	0.62	7.71	0.01	1.18	1.05	1.60
Location of contact with flu-like cases (2)	−0.74	0.98	0.57	0.45	0.48	0.07	3.24
Occupation	-	-	0.10	0.95	-	-	-
Student	−1.56	3.06	0.26	0.61	0.21	0.00	85.35
Preschool children	2.30	2.71	0.72	0.40	10.00	0.05	818.88
Age	-		0.63	0.43	-	-	-
7–14 years old (2)	1.74	1.91	0.83	0.36	5.71	0.13	242.28
3–6 years old (3)	2.33	2.76	0.79	0.40	9.97	0.06	818.88
Handwashing time	-	-	10.47	0.01	-	-	-
Handwashing time (1)	−2.88	1.13	6.57	0.01	0.06	0.01	0.51
Handwashing time (2)	−2.89	0.92	9.92	0.00	0.06	0.01	0.34
Covering mouth and nose when sneezing	0.29	0.67	0.19	0.66	1.34	0.36	5.02
Washing hands immediately after sneezing	−0.94	0.77	1.52	0.22	0.39	0.09	1.75
Handwashing after class	0.74	0.73	1.02	0.31	2.10	0.50	8.86
Thumb-sucking	-	-	0.25	0.88	-	-	-
Thumb-sucking (1)	−0.32	1.04	0.09	0.76	0.73	0.10	5.59
Thumb-sucking (2)	0.34	1.08	0.10	0.75	1.41	0.17	11.62
Rubbing eyes	-	-	0.42	0.81	-	-	-
Rubbing eyes (1)	−0.65	1.39	0.22	0.64	0.52	0.03	7.94
Rubbing eyes (2)	−0.37	0.67	0.32	0.57	0.69	0.19	2.55
Clean hands and nails (1)	−0.14	0.92	0.02	0.88	0.87	0.14	5.29
Mode of transportation	-	-	10.74	0.01	-	-	-
Mode of transportation (1)	−1.53	1.77	0.75	0.39	0.22	0.01	6.96
Mode of transportation (2)	−0.56	1.80	0.10	0.76	0.57	0.02	19.34
Mode of transportation (3)	2.60	1.00	6.83	0.01	13.47	1.92	94.75
Duration of stay	-	-	1.10	0.58	-	-	-
Duration of stay (1)	−2.86	3.49	0.67	0.41	0.06	0.00	54.11
Duration of stay (2)	−0.47	0.64	0.54	0.46	0.62	0.18	2.18
Wearing masks	−0.25	0.64	0.15	0.70	0.78	0.22	2.75
Covering mouth and nose when sneezing	−1.45	0.72	4.11	0.04	0.23	0.06	0.95
Ventilating by opening windows (<2 h)	3.33	1.55	4.59	0.03	27.99	1.33	589.54
Prophylactic medication	0.50	0.71	0.49	0.49	1.65	0.41	6.68
Sleep duration	−0.68	1.57	0.19	0.67	0.51	0.02	11.01
Frequency of colds	-	-	26.65	0.00	-	-	-
Frequency of colds (1)	6.05	1.50	16.23	0.00	424.73	22.36	8066.18
Frequency of colds (2)	2.89	0.68	18.36	0.00	18.04	4.80	67.74
Extracurricular training	3.48	1.19	8.57	0.00	32.55	3.16	335.29

## Data Availability

All data and statistical code to reproduce the results in the manuscript are available from the corresponding author upon reasonable request.
